# Requirements for an electronic handover system for interprofessional collaboration between psychotherapists and occupational health professionals – a qualitative study

**DOI:** 10.1186/s12913-022-08381-9

**Published:** 2022-08-25

**Authors:** Fiona Kohl, Peter Angerer, Lisa Guthardt, Jeannette Weber

**Affiliations:** Institute of Occupational, Social and Environmental Medicine, Centre for Health and Society, Medical Faculty, Heinrich-Heine-University Düsseldorf, Moorenstraße 5, 40225 Düsseldorf, Germany

**Keywords:** Mental health, Workplace, Occupational health, Handover, Psychotherapy, Communication

## Abstract

**Background:**

An electronic handover system provides a potential way to bridge the interface between psychotherapy and occupational health. This qualitative study therefore aimed assessing (1) content-related and (2) functional requirements that psychotherapists and occupational health professionals expect from an electronic handover system to exchange relevant information about their patients with common mental disorders.

**Methods:**

Five focus groups with psychotherapists and occupational health professionals (occupational physicians and members of company integration management) were conducted via video conference using an interview guide. The focus groups were transcribed and content-analysed using MAXQDA.

**Results:**

With regard to content-related requirements, information that serve to assess employee’s ability to work was described as particularly relevant by occupational physicians and members of company integration management (e.g. restrictions in certain work areas or ability to work under time pressure). Psychotherapists indicated that information about the employee’s working conditions is particularly relevant. This includes description of work tasks or conflicts at the workplace. Concerning functional requirements, all professional groups attached importance to data security and functions to improve communication and collaboration (e.g. the use of standardised handover forms).

**Conclusion:**

This study provides insight into the desired content-related and functional requirements by psychotherapists, occupational physicians and members of company integration management for an electronic handover system. However, the theoretical and practical development of such a system requires several additional steps, such as the involvement of further relevant stakeholders (e.g. patients, software developers).

**Supplementary Information:**

The online version contains supplementary material available at 10.1186/s12913-022-08381-9.

## Introduction

With a point prevalence of 16% of the world’s population, mental disorders are considered as a major global burden [1]. In Germany, an average of one in three women and one in five men suffers from mental disorders within a year [2]. The high prevalence combined with frequent reduction in work productivity and work ability as well as high absenteeism and risk of early retirement pose a medical and economic challenge [3–6].

In the treatment of employees with common mental disorders (CMD), collaboration between all involved health care actors is considered important to ensure comprehensive care as well as support and adaption of the working environment [4, 7–9]. In this context, collaboration with occupational health professionals is assigned particular relevance, as they could represent a crucial interface between psychotherapy and the employee’s workplace [7, 10, 11].

Occupational health professionals include various professionals involved in the care and treatment of employees such as occupational physicians (OPs) and members of company integration management (CIM). In Germany, OPs have either directly specialised in occupational medicine or followed a three-month occupational medical course and 9 months of practical training after acquiring specialist medical training within another field of direct patient care. The essential task of OPs is to advise employees and companies on occupational medicine issues. This includes, among other things, to perform risk assessments regarding mental and physical health, to deal with work-related health prevention, and to support employees in reintegrating into the workplace after sickness absence [12]. Occupational physicians therefore have a great deal of expertise in the prevention of work-related illnesses as well as knowledge of the employees’ working environment. In Germany, it is mandatory for companies to provide a company medical service [12]. CIM members are either specially employed for the tasks as CIM member or are employees (e.g. staff council) who belong to the CIM team in addition to their actual work [13, 14]. Goals of the CIM team are to avoid illness-related dismissals and to reduce absenteeism. Therefore, CIM members contact employees after a longer absence due to illness in order to discuss how reintegration into work could be possible. For this purpose, the employee’s workplace is analysed, the employee’s skills are compared with the requirements of the job and finally measures for job adjustments are developed [13]. Therefore, CIM members are seen as important occupational health professionals alongside OPs.

There is great potential in the diagnosis and treatment of mentally ill employees if OPs, CIM members and psychotherapists work together as a team. OPs can play an important role in diagnosing and treatment of mental disorders by identifying symptoms in employees and referring them for further care at an early stage [10, 11, 15, 16]. Previous research already indicated that psychotherapists would like to receive specific information about working conditions and work-related stress factors as problems at work are often a by-product or even cause or consequence of psychological stress. Such information could help psychotherapists to get a holistic picture of their patients and offer appropriate treatment [7, 10, 16–18]. Additionally, incorporating work aspects into treatment is considered successful in terms of a faster return to work [19]. Conversely, psychotherapists could support the employee’s return to work by exchanging information about the work ability to CIM members and OPs. This can, for example, prevent employees from being sent back to work with unrealistic expectations about work adjustments [20].

Although the need for collaboration has been acknowledged, previous research shows that OPs frequently encounter problems when trying to collaborate with different health care professionals [7, 17, 21, 22]. For instance, a previous study found that OPs often do not receive any information about their patients’ rehabilitation therapy or at least not until weeks or months after discharge [17]. A previous study illustrated that OPs are the professional group most likely to contact others due to a referral of a patient with a mental disorder. Conversely, they are rarely contacted by psychotherapists [7]. Among others, lack of time, missing contact details, low reachability and different working hours of OPs and other health care professionals are seen as reasons for the lack of cooperation [17, 23–26]. In a previous study, rehabilitation physicians stated that communication with OPs was difficult as coordination on a common patient often had to be done quickly and at short notice, which was not considered feasible with OPs [17, 26]. In order to reduce the time required for mutual information exchange, standardised communication forms have been proposed [26].

In order to improve handover and thus patient care, a number of standardised handover tools have already been developed [27–30]. In mental health care settings, however, studies showed that handovers are often unstructured and incomplete [29, 31, 32]. Accordingly, important information is frequently missing [31]. In occupational health care settings, previous studies already investigated the use of standardised handover tools to share information between OPs and attending physicians as well as rehabilitation physicians [33, 34]. The availability of standardised handover tools is associated with more frequent collaboration between OPs and attending physicians in the context of return-to-work and prevention of employees’ disease exacerbation [33]. Standardised discharge letters were used in one intervention study to improve communication between OPs and rehabilitation physicians in the treatment of patients with musculoskeletal disorders. In that study, OPs received these standardised discharge letters on average 2 days after patient’s discharge by the rehabilitation physicians faxing it to the OP or by handing it to the patient on the day of discharge [34]. In addition to standardisation of discharge letters, the authors of that study recommended an electronic transmission to avoid time delays due to hand over to the patient [34]. The use of an electronic handover system for interdisciplinary communication is also recommended by other researchers in order to ensure that information are provided quickly and easily accessible to involved health care actors [26, 32].

Deficits in mutual cooperation and information exchange are not only evident between psychotherapists and occupational health professionals, but also in various other interfaces in the health care system. For this reason, the use of electronic health records (EHRs) is being promoted internationally to improve collaboration in health care. In many countries, EHRs are already established, which means that patient information can be uploaded by physicians and other service providers [35, 36]. In Germany, the EHR has been introduced gradually since January 2021 [37].

So far, it is not known to what extent occupational health professionals can be involved in the use of EHRs. Additionally, it is unclear whether EHRs would meet the requirements of psychotherapists and occupational health professionals for mutual information exchange. The development of a specially adapted electronic handover system could improve cooperation between these professionals. For the development of an electronic handover system, it is elementary to consider the requirements of all involved stakeholders [38, 39]. When exchanging patient data of employees with mental disorders via an electronic handover system, a multitude of professional groups are involved. In addition to psychotherapists and occupational health professionals, these also include software developers, data protection officers and, in particular, the patients themselves, who might have the authority to decide on the handling of their data. The present study focuses on the requirements for information exchange between the professional groups involved at the interface of occupational health and psychotherapy.

The aim of this study was therefore to explore (1) content-related and (2) functional requirements that psychotherapists and occupational health professionals expect from an electronic handover system to exchange relevant information about their patients with CMD.

## Methods

### Study design and setting

This study is a subproject of a multicentre randomised controlled trial (RCT) testing psychotherapeutic consultation at work [40]. Within the framework of this RCT, the results of the present study should be used to develop and apply an electronic handover system for the exchange of patient data between OPs, CIM members and psychotherapists.

A qualitative study design was chosen as we considered it to be most appropriate for our research question. Qualitative studies are considered important for exploring and elaborating new ideas [41]. With regard to our research question, previous studies already focused in exploring obstacles in interprofessional collaboration and communication between OPs and other health care professionals [17, 26] Consequently, the use of an electronic software for interprofessional communication was recommended [26, 32]. However, to the current knowledge, there is no study that deals with concrete solutions for electronic information exchange in interprofessional communication between psychotherapy and occupational health. Conducting focus groups in a qualitative design allows us to discover and explore the views of stakeholders involved without the need of preconceived ideas through the researchers [41]. Furthermore, focus groups allow to observe common opinions by giving participants the opportunity to directly discuss each other’s recommendations [42]. Therefore, psychotherapists, OPs and CIM members were interviewed together during interprofessional focus groups. Focus groups were conducted between December 2020 and June 2021 as video meetings online by using the video conference platform “Cisco WebEx”. Focus groups were performed until data saturation was obtained. Data saturation seemed to be reached after four focus groups. However, a fifth focus group was conducted to confirm data saturation. Since this last focus group provided only little new information, the authors decided to stop further data collection. Each focus group lasted about 90 minutes. Allocation to the different focus groups was based on the scheduling preference indicated by the participants. The participants in the focus groups did not known to each other beforehand. The focus groups were conducted either during the participants’ working hours or after their working hours. Based on the research questions, a topic guide was developed by JW, FK and PA to ensure that all relevant topics are addressed by the focus groups. The topic guide was not piloted before the first focus group, but was sent for review in advance of the interviews to an external researcher who is very experienced in qualitative research and in the field of mental and occupational health. This topic guide (Additional file 1) started with an open introductory question about participants’ previous experiences of working with other professional groups in the treatment of employees with CMD. It further addressed what information could be exchanged between the different professional groups using an electronic handover system (i.e. content-related requirements) and which functions this electronic handover system should have (i.e. functional requirements). The focus groups were either conducted by JW or FK while Susan Gritzka (SG) or FK took field notes. FK has an educational background in physiotherapy and public health, whereas JW has an educational background in biomedical sciences and public health. Both team members have experience in occupational health research and are trained in conducting qualitative interviews through prior involvement in qualitative research or participation in specific university courses. All participants knew about the professional background of all team members.

Since the interviews were conducted in German, the quotes as well as the topic guide were translated into English by our co-author Lisa Guthardt, who has a professional background in English translation. To ensure that no meaning was lost in translation, she also reviewed the entire manuscript.

### Study participants

A purposive sampling strategy was chosen to recruit participants. Efforts were made to achieve a wide spectrum of opinions. To this end, participants with experience from different areas of the relevant professional fields, from different regions of Germany and with different levels of professional experience were recruited. OPs and CIM members were recruited as occupational health professionals from intercompany services and from companies of private and public sectors. Psychotherapists and psychiatrists working in outpatient and inpatient health care with more or less experience focussing on work-related problems were recruited. Study participants were reached through personal contacts of the research team and through an education course for occupational medicine. For all occupational groups, inclusion criteria was having at least 1 year of professional experience in their work as a psychotherapist, OP or CIM member and sufficient knowledge of German to be able to conduct the focus groups in German. No other inclusion or exclusion criteria were applied. A total of 81 health care professionals were invited to participate in the study (45 personal contacts, 11 participants of the education course) by sending E-mails with a standardised information letter. Of these, 32 health care professionals agreed to participate. Seven participants were unable to attend the focus groups due to scheduling conflicts (*n* = 5) and illness (*n* = 2). In total, five focus groups were conducted with 25 participants (11 occupational physicians, 9 psychotherapists/psychiatrists, 5 CIM members). The aim was to include two representatives from each professional group in each focus group to ensure even distribution. The first three focus groups were conducted with participants of all three professional fields. After major concerns about sharing information with CIM members had been expressed in these focus groups, the last ones were conducted with psychotherapists and OPs only.

As also personal and professional contacts participated in the study, the interviewers FK and JW knew some of the participants.

Additional information on all participants regarding sex, age, education, occupation and work experience were collected using standardised questionnaires.

### Data analysis

All focus groups were audio-recorded and transcribed. Subsequently, the transcripts were content-analysed [43] by FK using the MAXQDA 2018 software package. First, (1) content-related requirements and (2) functional requirements were included as overarching categories by deductive coding. Content-related requirements were further deductively categorised in (i) information for occupational physicians, (ii) information for psychotherapists, and (iii) information for CIM members. These overarching categories were subdivided into subcategories by inductive coding. During inductive coding, relevant text passages in the transcript were first color-coded and then paraphrased. From these paraphrases, an initial category system was created and implemented in MAXQDA. To ensure that one author’s personal and professional attitude did not influence the analysis, analyses were conducted by two authors. After coding the first focus group, JW reviewed the coding system and provided suggestions for adjustment to FK. Then, all focus groups were analysed by using the adjusted coding system. In a sec ond round of review, JW checked the coding system again and discussed possible changes with FK. Since this second review round only led to a few adjustments, FK conducted a second and thus final coding round.

Field notes documented when participants were temporarily unable to participate in the focus group or were seen without a picture (e.g., due to internet problems). This information were used to ensure that there were no biases in the results due to such inconsistencies.

Transcripts were not given to participants for comments or corrections due to logistic constraints.

The completed checklist of consolidated criteria for reporting qualitative research (COREQ) [44] can be found in Additional file 2.

## Results

### Study population

Characteristics of the study population are shown in Table 1. The majority of participants were over 40 years old (84%) and female (72%). Overall, occupational physicians and psychotherapists showed a wide range of professional experience. The professional experience of CIM members was overall lower than in the other occupational groups. Most of the OPs were also members of CIM teams (73%). Among psychotherapists, one participant was also a CIM team member.

**Table 1:** Characteristics of the study population (*n* = 25)

	Psychotherapists	Occupational physicians	CIM members^a^
n	9	11	5
Age in years^b^	54 (33–67)	57 (36–68)	49 (42–61)
Female gender, n	4	9	5
Years in current job^b^	15 (2–40)	20 (2–34)	15 (2,5–18)
Active in CIM team, n	1	8	5
Years active in CIM^b^	10 (10)	5 (1–10)	5 (2,5–9)

^a^CIM = Members of company integration management

^b^Data are presented as median with min-max

### Results from the focus groups

**Fig. 1:**
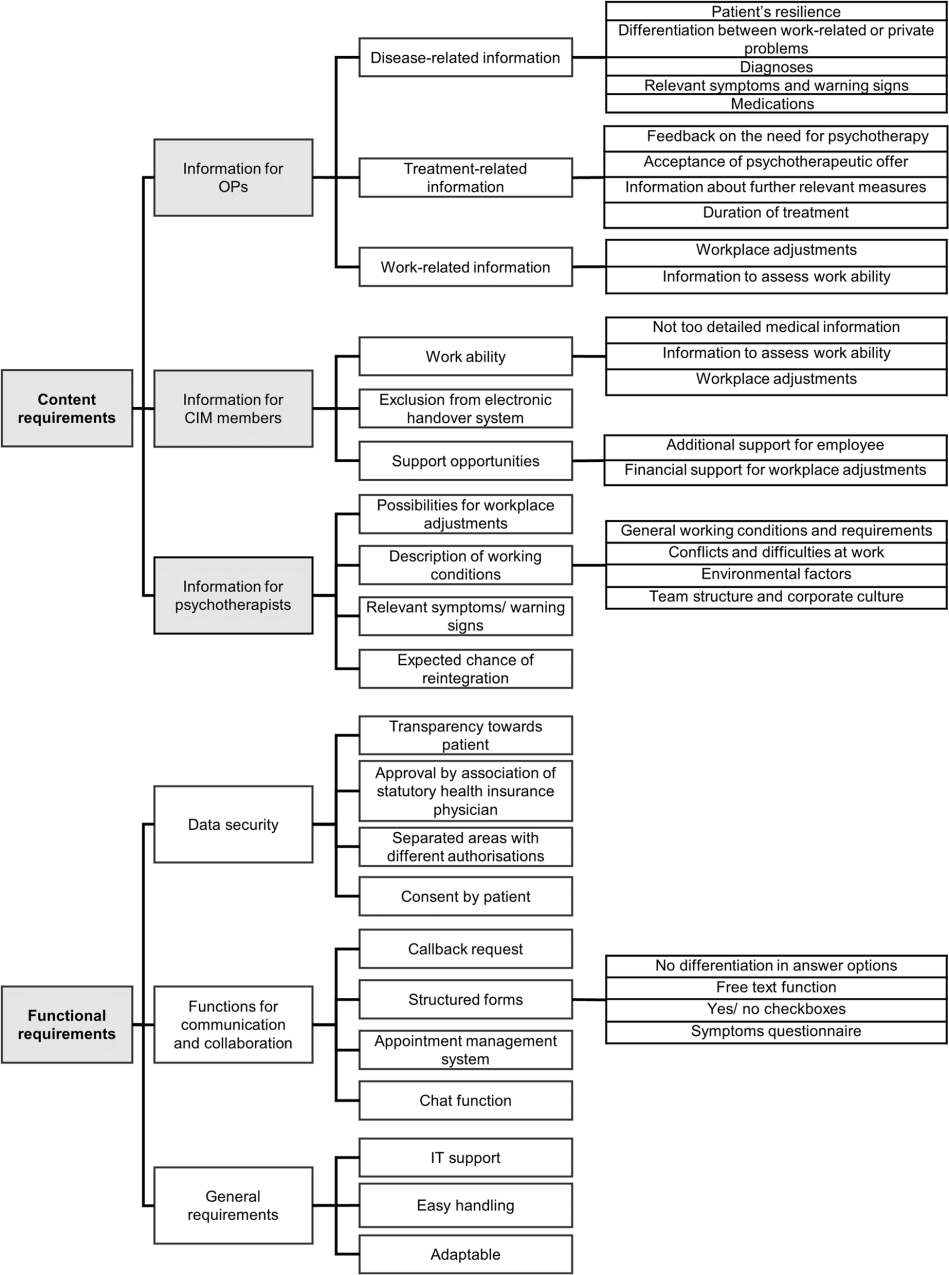
Category system of transcripts. Deductive categories are highlighted in gray, inductive categories are highlighted in white

The category system is displayed in Fig. 1.

#### Content requirements

The relevant quotes can be found in Table 2. In the following chapter, the quotations contained in Table 2 are given in the text to the corresponding text passages (e.g. QO1).

**Table 2:** Content requirements – categories and exemplary quotes

**Information for OPs**	
**Disease-related information** O1: What is important for me is some kind of description of the resilience: Is he even resilient yet? And where does he have potential deficits? Because this is something I can include in the work profile and I could also say: Okay, he can’t do that yet or at least not to that extent or we can just leave this work step out. Please find him another task. (OP, FG2) O2: And I also want to know about his medication, for example. Does he have any concomitant medication over the next half year that might restrict him in doing shift work, for example? […] Or wheresoever. Or in the power of concentration. (OP, FG2) O3: I’m also concerned about concrete diagnoses, which I have to find out from psychotherapists, because for us, it’s also important to know […] why the occupational physician contacts us. Because sometimes, the employer has the option to commission a report from [an organization], if it’s about occupational suitability, and then, it really matters if someone has a psychosis or an addiction, which is why we need to know about concrete diagnoses as well. (OP, FG1) O4: It’s especially difficult with bipolarity, because it has to do with quick mood changes that doesn’t always seem to be plausible. […] But it’s only possible to develop a good management if the occupational physician knows who to contact, especially when there are quick changes of mood, phase changes. So that you can even talk about warning signals. But that really requires a trustful collaboration. But if that works, the prognosis for people with bipolarity often improves significantly. (Psych, FG2) O5: It might be a good idea to separate underlying problems of the current situation; you could separate them in private-related and work-related problems. Then you would already have some sort of sounding, you would get such feedback. Because, well, we can’t do much about private-related problems as occupational physicians. […] But we can actively tackle work-related issues. (OP, FG5)	
**Treatment-related information** O6: I would really appreciate getting more information on, let’s say, estimated duration of therapy, you know? So that I roughly know when an employee can be expected to return to work; or maybe when the end of therapy can be expected so that I might be able to plan further measures like rehabilitation. (OP, FG4)	
**Work-related information** O7: And like I said before, it would be nice to have some kind of prognosis, as to whether certain continuing restrictions concerning specific work areas can be expected or whether there will be some straining situations. (OP, FG4) O8: Yes, questions are often related to the ability to do shift work, so whether day shifts are the only option or whether alternate and night shifts are also possible. (OP, FG4) O9: Then, working under time pressure, deadline pressure, conflicting activities, such information is also requested there and it’s also helpful for the assessment or the evaluation, to know if someone can still continue corresponding tasks, in full shift or even part-time. (OP, FG4) O10: But I would need to have a given reintegration proposal plan including all the points mentioned: How many working hours, duration of the measure, shift work, concentration, (approximately the ideal) resilience, medication, special conflict situation? (Psych, FG2) O11: So, performance capacity, and prognoses about whether working under time pressure or under deadline pressure is possible. (OP, FG4) O12: That I receive a written report [any report by the psychotherapist] […] that leads to further questions, specific questions, depending on the kind of action. (OP, FG4) O13: Therefore, I think that an exchange between attending physicians and occupational physicians is extremely important. And I’d like to do it electronically, as well. And then, we can perform an adequate assessment of the work ability, for the CIM team for example. (OP, FG2) O14: We mustn’t overload psychotherapists with what kind of information they can provide. Right? If they don’t know the operational processes, it’s not really useful to write down: no shift work, for example. You know? And they don’t know whether that’s an exclusion criterion and if the employee might not be able to work anymore at all then, because he is doing shift work and there’s no other option. (OP, FG2) O16: I think people’s expectations on what would be of help might be useful, like physician 2 also said. And also, what do I interpret into my patients, how is their perception of the workplace? How do they perceive it? Do they feel somehow restricted or pressured? What is really necessary in terms of operational concerns that can’t be changed? Und that’s why they don’t say anything at all, or they say something and feel like they’re running into a wall. For example, when it comes to shift schedules or things like that. (Psych, FG 5)	
**Information for CIM members**	
C1: I think that such an electronic patient file, this exchange of information, must stay between physicians. So, between external occupational physicians, yes. Involving the internal CIM team, for example we have members of the works council in the CIM team. We have people in there who have been called in by the employer. If you provide them with information covered by medical confidentiality, if you include them in this circle, then/ There are so many concerns about individual data in a company, which information is known to whom, that the options of who can be included in the CIM team by the company are very limited. (CIM, FG2)	
**Work ability** C2: How does a problem-compatible workplace look like? And what are our options as employers to enable such a workplace? And is it (even) possible to enable it within the scope of our possibilities? (CIM, FG2) C3: How can that person be deployed, for example firefighters after having PTSD or else, how can we make this work, how can we lead someone back to work, and I would really like to see more support from attending psychotherapists, because works council consists of occupational physicians, and from a nonmedical, unprofessional point of view, I can say that this is certainly one facet, but the therapeutical aspect would be incredibly important. (CIM, FG1) C4: I think a detailed description would be very important, too: What is feasible for the employee? And what’s not? You shouldn’t neglect one of the two aspects, always explain both: What is not feasible? It’s often said, no shift work. But what does that mean? Is it early shift, late shift or night shift? We don’t have night shifts at all, we only have late shifts. But what exactly does that mean? (CIM, FG2) C5: What is also important is that concrete causes and diagnoses shouldn’t be mentioned to the employer right away. Instead, we want to focus on restrictions. So there can be many causes or diagnoses, but they always lead to the same restriction. (CIM, FG3)	
**Support opportunities** C6: And when talking about concrete options of adjustments, one question often arises, because this is usually associated with immense costs. Is it possible to receive some support? Something like integration offices, like special services on integration elsewhere. Could we get an attendant for the employee? (CIM, FG2)	
**Information for psychotherapists**	
**Description of the workplace situation** P1: Occupational physicians could provide concrete information about the workplace and all requirements, for example is it shiftwork or cycle-related work. This could be really important for psychotherapists. (OP, FG1) P2: I think it would be very important to know about how things are handled in the company or how the corporate culture is. (Psych, FG1) P3: It’s important for us to be aware of stress factors at work, and these are not only environmental aspects like chemicals that can affect the psyche as previously mentioned, but also structural aspects, how is the team structured, how do people work together there. (Psych, FG1) P4: Let’s say bullying for example or stress at work that has nothing to do with the private environment. You would probably need to have some background information from the occupational physician. (OP, FG5)	
**Expected chance of reintegration** P6: For example, we have a cooperation where a CIM member sends patients to us, and he also coordinates implementations if necessary, and if he says that we can no longer employ the employee anymore, that all options are exhausted, it’s a completely different frame for me as a therapist and that’s really helpful for my work. (Psych, FG1) P7: We were talking about his reintegration, and I said that it might be exhausting for him to start full-time because his sick leave lasted for quite a long time. And he explained to me that he preferred not to do that as long as he didn’t get more money. Luckily, he agreed that I can talk to his resident psychotherapist, whom I also knew. And she herself also said, yes, she thinks that she has to discuss that with him, too. (OP, FG2)	
**Workplace adjustments** P8: Sometimes, we also have some more questions, for example which adjustments might be possible at the workplace. (Psych, FG4)	

*OPs* Occupational physicians, *CIM* Members of company integration management, *Psych* Psychotherapists, *FG* Focus group

#### Information for occupational physicians

From the participants ‘perspective, electronic transmission of disease-, treatment- and work-related information to OPs is desired to ensure comprehensive assessment of employee’s work ability.

##### Disease-related information

OPs stated that they wish to receive information from psychotherapists about patient’s mental condition. This includes a description of the *resilience* in order to be able to assess stress limits and deficits at work (Quote (Q) O1). Information about *medications* taken by the employee was indicated as relevant to judge whether these medications lead to limitations in the ability to work (QO2).

Transmission of *diagnoses* was mentioned as important as these are needed to draw up vocational suitability reports for the patient’s employer (QO3).

According to participants, it would be helpful to exchange *warning signs and relevant symptoms* between psychotherapists and OPs via an electronic handover system to improve management of mental disorders and their prognosis. Such warning signs include mood changes in bipolar disorders or suicidal thoughts (QO4).

It was expressed that it would be helpful if psychotherapists could give feedback to OPs after the initial consultation as to whether patient’s complaints were based on *work or private-related problems*. This might help OPs to evaluate whether they should contact psychotherapists (e.g. to exchange information about workplace adjustments) (QO5).

##### Treatment-related information

It was mentioned that some information about the psychotherapeutic treatment process should be exchanged via an electronic handover system to assist OPs in their patient management. Information regarding *duration of therapy* would allow OPs to estimate when an employee can be expected to return to work. In addition, OPs would like to receive information about *further measures* and steps in the treatment process in order to initiate necessary further treatment at an early stage (e.g. rehabilitation clinic) (QO6). Furthermore, OPs indicated that they would like to receive feedback from psychotherapists about whether psychotherapy was indicated for the patient referred and whether the patient accepted the therapy offer.

##### Work-related information

OPs expressed that they would like to receive the following information from psychotherapists to assess the employee’s current and future work ability (QO7-QO13):

1. Restrictions on work areas and tasks
2. Ability to do shift work
3. Recommended number of working hours
4. Ability to concentrate
5. Estimated resilience of the employee
6. Ability to work in conflict situations
7. Ability to work under time pressure and deadline pressure
8. Ability to work with customers

However, concerns were also raised that psychotherapists may be overburdened with those requirements if they are not sufficiently familiar with the employee’s workplace and work routines. Furthermore, participants feared that some assessments could jeopardise a job if psychotherapists’ recommendations are not feasible (QO14). Therefore, it was expressed that mutual exchange between OPs and psychotherapists is essential to ensure a well-founded assessment of work ability (QO13).

OPs indicated that they would like to receive information about recommended *workplace adjustments* via an electronic handover system (e.g. patient expresses a wish for a single office during psychotherapeutic consultation) (QO15).

#### Information for CIM members

The electronic exchange of medical patient information with CIM members was controversially discussed within our focus groups. In all occupational groups, some participants would refuse to exchange information with CIM members if it involved information being covered by medical confidentiality (QC1). The content-related requirements of participating CIM members who would welcome the use of an electronic handover system are described below.

##### Work ability

It was several times expressed that CIM members would like to receive information relevant for evaluation and promotion of work ability. In this context, participating CIM members perceived joint exchange with OPs and psychotherapists to be significant. It was expressed that it would be important to explore which adjustments the employer is able to make within the company’s capabilities (QC2). Support from psychotherapists was expressed as desirable to obtain information about possibilities of reintegration (e.g. firefighters after post-traumatic stress disorder) (QC3). Additionally, it was expressed that psychotherapists should make their statements about work ability and workplace adjustments as specific as possible to the individual employee’s workplace (e.g. “no night shift” instead of “no shift work) (QC4). Furthermore, CIM members pointed out that they would not like to receive detailed medical information (e.g. diagnoses) as it is not considered relevant for their work (QC5).

##### Support opportunities

CIM members indicated that they would like to receive information about various support options, for example through integration services or financial support options for the employer to adapt the workplace (QC6).

#### Information for psychotherapists

##### Description of working conditions

Electronic exchange of information about the employee’s working conditions was considered to be important for psychotherapists. More specifically, the following aspects were mentioned: (1) general working conditions and requirements (e.g. working hours, work areas and tasks, shift work (QP1)), (2) team structure and corporate culture (e.g. how the company deals with mental disorders (QP2, QP3)) (3) environmental aspects (e.g. working with psychoactive chemicals (QP3)), (4) conflicts and difficulties at work (e.g. bullying, psychological pressure at work (QP4)).

##### Expected chance of reintegration

The participating psychotherapists indicated that it would be very helpful to know how the company feels about the employee’s reintegration (e.g. chance of continued employment) (QP6). Furthermore, it was expressed that occupational health professionals could inform psychotherapists if changes in employee’s job security occurred during the course of the treatment (e.g. because the employee shows no motivation to reintegrate) (QP7). Both aspects would allow psychotherapists to adjust their therapy accordingly.

##### Possibilities for workplace adjustments

Psychotherapists would like to receive information about possibilities to adjust the workplace to the employee’s health condition. This includes adjustments that are already planned in the company and feedback on whether recommended adjustments by the psychotherapist are possible to implement in the company (QP8).

#### Functional requirements

Relevant quotes for functional requirements can be found in Table 3. In the following chapter, the quotations contained in Table 3 are given in the text to the corresponding text passages (e.g. QF1).

**Table 3:** Functional requirements – categories and exemplary quotes

**Data Security:** F1: You [person’s name] have already mentioned data security, but from my experience I can tell that it’s very important. Affected persons are often worried about some kind of stigmatisation and they are concerned that information is leaked and spread in the company, so you have to provide confidence-building measures, that’s the most important aspect at this point. (OP, FG1) F2: I would like to give another example: During the corona pandemic, there were many challenges concerning therapy sessions being offered online/ and many patients were reluctant and concerned that data protection was violated […] and what I found very helpful to increase patient trust was that you were only allowed to use systems that had been approved by the Association of Statutory Health Insurance Physicians, because people generally trust such a system. (Psych, FG1) F3: Yes, I think it’s best if such a system is not located at the employer but rather externally, so that you can really assure the patient that you are only involved as an occupational physician because you are responsible for the person and that this is not related to the employer. (OP, FG1) F4: And it must be completely transparent for the employee who has access to the data and to what extent. (OP, FG4) F5: I think a general area for almost everyone would be important and certain restricted areas where patients could actively give access to selected people if desired.. (CIM, FG1)	
**Functions for communication and collaboration** F6: In the context of the quickly advancing digitalisation […] I have to say that there’s a huge advantage of this chat-function, you know? So that you have a programme that shows notifications, and you don’t have to start the e-mail programme, for example. This is then also related to the encoding of messages, isn’t it? […] So that you have a separate IT platform that enables a secure chat function, for example. This would be the best idea, I think. You could quickly send someone a short message. I often experience that you don’t have time to immediately answer the phone or that the other person doesn’t because they are doing a treatment or are busy otherwise. A chat function would solve this problem as you could just send a short message: Listen, we need to talk about this topic again. When can we talk on the phone? I think this always works better than sending three e-mails back and forth. (OP, FG4) F7: We were actually using a management system for appointments. […] This means if both parties agree via management-system/ every Monday at 11 a.m., there is a free hour and you can write down a [appointment with a] therapist or someone belonging to the occupational physicians with my cooperation partner, this already helps quite a lot. […] And they customise the systems, for example, so they also make sure that patients are reminded the day before and so on. (Psych, FG2) F8: It’s similar to team coordination. I think it’s really good, also concerning implementation, to make everything more transparent and to be able to display what is achieved, achieved in the CIM team and along with the employee in order to restore one’s health. Unfortunately, it’s very difficult for me to implement it, when you have so many company sites and you have to work with many occupational therapists and there is no contact person or the CIM team cannot do it to this extent. (CIM, FG2) F9: I would also do it like this. You should be able to simply tick the basic aspects. Night work, shift work, all these things. You might also mention current work times per day, six hours or nine hours, eight hours, you name it. That you could also have small spaces for short notes. But mainly checkboxes with the most significant aspects. And also room for free text where you can describe the current individual symptoms so that you have a sheet, some kind of referral, with the most important information. (OP, FG5) F10: Especially for these cases, you could also use the BDI-II, for example, which has 20 questions like this, it can be completed within 10 minutes, or there is also the Symptom checklist 90. It covers general psychiatric symptoms. It’s a longer questionnaire that could be filled out by the employee, and you could implement the results in such a system, and you could detect where the standard value is exceeded. (OP, FG1)	
**General requirements** F11: It is necessary to have a responsible IT support who can be contacted and who takes care of possible issues. We are currently experiencing that with our software. No support, no one. Nobody is responsible. Nobody customises the software. It’s horrible. (OP, FG1) F12: So, it has to be smart, and it has to be adaptable, I can speak from experience. We might need something completely different in 5 years, so it shouldn’t be a fixed product that can’t be changed easily, for example, according to different legal requirements or stakeholders’ requirements. I would really pay attention to this, that you tell the IT provider to “keep it simple”, but to make it easily adaptable at the same time. (OP, FG1) F13: It should be intuitive. This means that it should be simple in operation, so that you can use it with few instructions and without any training. (Psych, FG1)	

*OPs* Occupational physicians, *CIM* Members of company integration management, *Psych* Psychotherapists, *FG* Focus group

##### Data security

Study participants mentioned a high level of data security as an elementary requirement to protect sensitive patient data. Data security was also mentioned as a prerequisite to gain patient trust in the system (QF1). One psychotherapist suggested that an approval of the electronic system by the Association of Statutory Health Insurance Physicians would promote patient trust (QF2). Moreover, one OP expressed that an electronic handover system should not be managed and located by the employer but by an external and independent organisation (QF3).

It was mentioned that transparency about all user actions in the system should be ensured to the patient. This includes transparency about who has access to data and which data is exchanged (QF4). Furthermore, it was expressed that patient consent to share information should be technically implemented into the system. This would allow patients to decide who can see what information. In this context, it was suggested that patients actively grant individuals access to certain areas (QF5).

##### Functions for communication and collaboration

Study participants reported that telephone contact between psychotherapists and OPs is often difficult as work schedules can vary greatly or OPs often work on the move. Therefore, they would welcome a secure *chat function* for short messages to exchange information or arrange an appointment for a telephone call (QF6).

One focus group discussed that an *appointment management system* has the potential to facilitate cooperation. They proposed a calendar function showing available time slots (e.g. for phone calls) in which all stakeholders could enter appointments with each other. They further suggested that patients could also be integrated into this system and receive reminders for arranged consultations (QF7). Members of the focus group argued that an appointment management system would facilitate transparency about joint collaboration, but they also expressed concerns that working with an appointment management system would be challenging if CIM members were responsible for multiple company sites and worked with multiple OPs (QF8).

##### Standardised forms

Study participants expressed that an exchange of information using *standardised forms* in the system would be a helpful option. That way information on working conditions, conflicts at work and patient’s work ability could be exchanged between OPs and psychotherapists. The forms should be designed in a way that yes/no checkboxes can be ticked (QF9). Furthermore, it was mentioned that standardised instruments could be used to share information about relevant symptoms (e.g. with validated mental symptom questionnaires) (QF10).

##### General requirements

One OP highlighted that an electronic handover system needs responsible IT support who is available and can fix problems (QF11).

It was further stated that the system should not be fixed. Instead, it should be adaptable to possible changes in legal requirements or stakeholder requirements (QF12).

It was emphasised that the system should be simple in operation, intuitive and should not require any training to operate it (QF13).

## Discussion

In the present study, participants identified several content-related and functional requirements for an electronic handover system to exchange information between psychotherapists and occupational health professionals. With regard to content requirements, psychotherapists stated that they wish to receive information about employee’s working conditions, the likely chance of reintegration into the company and possibilities to adjust the workplace to the employee’s health condition. OPs and CIM members were interviewed as occupational health professionals who particularly desire an electronic information exchange with psychotherapists in order to assess the employee’s work ability. Concerns were expressed about electronic information exchange with CIM members regarding medical information being covered by medical confidentiality. With respect to functional requirements, functions related to data security, mutual communication and cooperation as well as general requirements for the practicability of the system were proposed.

In our study, there was a controversial discussion about the extent to which psychotherapists can and should provide occupational health professionals with information about the employee’s work ability. These results are in agreement with those obtained by a previous study in which expectations of OPs towards psychotherapists regarding reintegration of an employee with CMD were determined [20]. On the one hand, OPs in that study wanted psychotherapists to provide concrete information about the employee’s work ability, but on the other hand, they also feared that jobs could be endangered if too many work restrictions were indicated [20]. In addition, that study pointed out that psychotherapists should not create false expectations in patients about possible changes in the company concerning adaptations of the workplace. However, the need to consider necessary changes in the workplace is underlined by another study [10]. In that study, it was expressed that in case psychological stress is related to working conditions, the success of psychotherapeutic counselling is dependent on whether necessary workplace changes are carried out. Accordingly, participants of our study emphasised the importance of a mutual information exchange between psychotherapists and occupational health professionals. They argued that psychotherapists are only able to provide reliable information about the work ability if they are sufficiently informed about the employee’s working conditions. Therefore, psychotherapists in our study indicated that they would like to receive information on the employee’s workplace and difficulties at work. These results are in line with previous studies [7, 18, 20, 26]. In our study, some new aspects on this topic emerged as participants expressed further specific wishes, e.g. concerning team structure or environmental aspects that might affect employee’s mental health.

In addition, new topics emerged in our study. Participating psychotherapists stated that they would like information about expected chances of reintegration and possibilities to adjust the workplace to the employee’s health condition in order to use this information for therapy planning.

OPs in our study stated that they would like to receive from psychotherapists information to assess employee’s work ability (e.g. expected limitations at work) as well as information that would support OP’s further treatment planning (e.g. planning for rehab or expected return to work). Previous studies support our results [17, 23–26]. However, these studies expressed that barriers such as too little time inhibit mutual cooperation. This time aspect might be improved by developing an electronic handover system for information exchange.

Furthermore, it was expressed in our study that OPs would like feedback on whether psychotherapy was indicated for the referred employee. In another study, it was reported that some employees were not referred by involved occupational health professionals for psychotherapy despite psychological symptoms. A possible reason given was that the occupational health professionals involved felt that the employee would not benefit from it anyway [10]. Feedback that psychotherapy was indicated and successful for the employee could encourage occupational health professionals to continue to identify and refer employees with CMD.

Another aspect controversially discussed in our study was the participation of CIM members in an electronic handover system. Since a CIM team does not only include OPs but also professionals from the staff council, some participants had concerns about sharing medical information electronically with CIM members. CIM members are subject to confidentiality, which is, however, not equal to medical or therapeutic confidentiality. Nevertheless, a systematic review found that employees with CMD think that cooperation between stakeholders involved at the workplace and rehabilitation would promote work participation [45]. In addition, this review indicated that colleagues believe that it is the employer’s responsibility to contact the rehabilitation stakeholders to receive information on how to manage employees with CMD. In our study, possible solutions were proposed in terms of data security, so that different involved professionals can only see the content that is relevant to them. In this context, it could be re-evaluated whether CIM members should become part of an electronic handover system if it is technically ensured that they do not have access to information that are subject to medical confidentiality.

Data security was mentioned in our focus groups as an elementary prerequisite for information exchange. It was desired that the electronic handover system provides different access areas to ensure that the professionals only receive information being relevant and intended for them. Our participants further suggested that the patient could actively give access to these areas to the different professionals. This would give the patient a central user role within the electronic handover system. This user role is comparable to the patient role in EHRs in health care systems of various countries. Currently, some EHRs already allow patients to grant access rights to family members or other external actors [36]. In Germany, the law strictly regulates who might have access to the EHR including physicians, therapists, pharmacies and other service providers involved in patient care. However, also in the German EHR the patient is still able to manage access to uploaded information to those health care providers [37]. The participation of CIM members in the German EHR still needs to be discussed.

In our study, it was also emphasised several times that it should be transparent to the patient which information is exchanged with whom. The Austrian EHR „ELGA“, for example, displays all movements in the system to the patient [36]. Although transparency to the patient does not exist in all EHRs, it is standard at least in the EU [36]. In Germany, Section 630 g of the German Civil Code stipulates that patients should be given access to the complete patient file upon request [46]. There are only restrictions in this right if, for therapeutic reasons, access to sensitive data could lead to a significant health risk of the patient.

With regard to the exchange of such sensitive data, one might discuss the use of a chat function as it was desired by participants in our study. This chat function could additionally replace a telephone conversation or could be used to arrange a telephone appointment. Secure chat functions are already part of some EHRs, and previous studies demonstrated that they are readily used for interprofessional information exchange in diabetes and primary care [47, 48]. Various companies have already developed solutions to securely send messages and medical reports (e.g. X-ray images) between different health care professionals as well as with patients and relatives [49, 50]. The respective legal requirements in the individual countries determine the extent to which content exchanged via a chat function within an electronic handover system must be stored and disclosed to the patient.

In order to improve mutual cooperation, participants of our study suggested integrating a solution to manage appointments within an electronic handover system. With the help of an appointment management system, free capacities of the involved health care professionals could be visible and appointments such as telephone calls could be booked. Similar approaches already exist regarding management of patient appointments [36, 51, 52]. To the best of our knowledge similar solutions for collaboration between health care professionals still need to be developed.

Consistent with previous research [26], participants of our study stated that the use of standardised forms could support information exchange. According to participants, these forms should comprise a combination of predefined questions (e.g. shiftwork yes/no) with check boxes and free text fields. In our study, the use of standardised forms for exchanging information about employee’s work ability was mentioned as one example. Previous research has shown that the work ability of patients with depression is assessed differently by different therapists [53]. Therefore, the use of a validated MINI ICF APP instrument is recommended to compare the employee’s ability profile with the job requirement profile [54, 55]. The use of such validated measurement instruments could also be discussed for the exchange of further content requirements mentioned in our study.

Participants in our study further expressed some general requirements for an electronic handover system. These mentioned requirements are generally considered to be among the most important principles of user-friendly software development [56]. When developing an electronic handover system, these aspects should be taken into account.

### Strengths and limitations

One strength of our study was the interprofessional composition of the focus groups, which enabled an exchange between the different professions. Conversely, if participants had concerns regarding information exchange with the other professionals, they may have expressed themselves less critical due to the interprofessional composition. However, since numerous critical statements were made by all professional groups about information exchange with CIM members, even though they were part of these focus groups, it can be assumed that participants were not inhibited in expressing their opinions.

Participants were recruited from different areas of the respective professional group (i.e. OPs and CIM members from private and public sectors, psychotherapists from outpatient and inpatient sectors) and comprised a wide range of age and professional experience to maximise the likelihood of obtaining a sample with a wide range of perceptions. However, it must be mentioned that the gender proportion was unevenly distributed in the different professional groups. Overall, 72% of our participants were female (82% among OPs, 44% among psychotherapists, 100% among CIM members). Consequently, perspectives of female participants may be overrepresented among OPs and CIM members and underrepresented among psychotherapists [57].

The focus group interviewers knew some of the participants. To ensure that no distinction was made between known and unknown participants, all participants were sent the same information in advance (information sheet). However, we cannot exclude the possibility that this prior familiarity may have had an impact on the trust relationship between participants and interviewers [58]. This could have had an influence on whether, for example, more or less critical statements were made regarding the development of an electronic information system or cooperation between the professional groups.

The authors of this study are also working in the multicentre RCT for which the results of the present study will be used to develop an electronic handover system. We have made every effort to be objective and have given the topic guide to an experienced person in the field of qualitative research for review in advance. Nevertheless, it cannot be entirely ruled out that the analysis and interpretation of the results may have been biased to be advantageous for use in the RCT. However, FK and JW, who conducted the interviews and analysed the data, do not belong to any of the professional groups interviewed. Therefore, when conducting the interviews and analysing the data, they did not include their own experiences regarding interprofessional communication.

Due to concerns about electronic exchange of medical information with CIM members, two focus groups were conducted without CIM members. Therefore, data saturation may not have been achieved in this professional group. However, as most of the OPs were also members of CIM teams, it can be assumed that the perspective of CIM members was adequately described.

Unfortunately, for logistical reasons, it was not possible for us to send the transcripts to the participants for correction. Therefore, it cannot be ruled out that the participants wanted to expand or change aspects before the analysis.

Due to the Covid-19 pandemic, focus groups could only be conducted online. This may have had a negative impact on the dynamics of the conversation, as aspects of a face-to-face conversation were missing.

### Implications

The use of an electronic handover system for interprofessional information exchange has already been recommended in previous studies [26, 32, 34]. In our study, content-related and functional requirements for an electronic handover system were presented. The results of our study could be used to develop a quantitative study design. With the help of quantitative studies and larger study samples, our results could be extended and validated. Furthermore, future studies might analyse the controversial aspects discussed in our study. Electronic information exchange of patient medical information with CIM members was rejected by some of our participants. The comparison with EHRs shows that electronic systems allow subdivision into various areas with different authorisations. With regard to this aspect, the extent to which CIM members should be involved in an electronic handover system could be re-examined in further studies.

In our study, as well as in previous studies, it was found that the use of standardised forms for information exchange is desired. Future research might therefore investigate to what extent content requirements presented in our study are already covered by existing measurement instruments and whether those instruments could be integrated into an electronic handover system (e.g. MINI ICF APP instrument for information exchange about work ability [54]). Furthermore, the content requirements presented in our study could be used to develop further standardised forms.

The focus on interprofessional communication between occupational physicians, CIM members and psychotherapists used in this study is very specific and therefore restrict transferability to other settings. However, disease-, treatment- and work-related information for OPs and information about work ability for CIM members might also be important when treating patients with other diseases. Similarly, information about the patient’s workplace could also be useful in treatment in the context of other specialties, such as musculoskeletal rehabilitation [59]. With regard to structural requirements, the results of this study are less specific and can probably also be applied to communication between other medical professions. Nevertheless, complementary research consulting professional groups of other medical fields is recommended to assess their specific content and structural requirements for electronic handover.

Additionally, further analyses are necessary to examine whether the functional requirements presented in our study could be implemented in already existing EHRs (e.g. in the form of uploading a report of medical findings).

The development of an electronic handover system is one possible solution to improve interprofessional communication between occupational health professionals and psychotherapists. The results of our study represent one step in the development of such a system. However, the extent to which stakeholders who would use such a system believe it would truly facilitate handover would need to be examined in a larger and representative sample. Furthermore, some concerns about collaboration with OPs have been raised in previous studies regarding lack of confidentiality or favouring employer’s interests over patient’s ones [17, 60]. In our study, concerns were mainly expressed about sharing information with CIM members. We made some suggestions on how to address these concerns. For example, different areas could be set up so that not all stakeholders have the same access rights. Also, patients could specify exactly who should receive what information. Therefore, further research should be conducted to determine whether an electronic handover system for interprofessional communication would be used by all stakeholders, taking these aspects into account. Concerns in this regard would need to be taken into account in further development.

The results of this study should not be seen as a manual for the development of an electronic handover system but rather as an insight into the requirements mentioned by potential users. Furthermore, other stakeholders being involved in electronic information exchange need to be interviewed about their requirements. This include patients, who would play a crucial role in the use. Patient option is particularly important because a fundamental requirement for an electronic handover system is the patient’s consent for information exchange. Furthermore, many open questions arise from the results of our study, which not only have to be examined with regard to technical implementation possibilities, but also with regard to legal requirements. For example, it has to be checked how a chat function could be integrated into an electronic handover system, which content could be shared via a chat function and to what extent this information would need to be stored. To answer those questions, as well as for the further development of an electronic handover system, it is highly relevant to involve data protection officers, software developers and other key stakeholders.

Finally, conclusions of the theoretical approaches could be used for a practical development and implementation of an electronic handover system.

## Conclusions

In interprofessional communication, the time factor in particular is cited as a limiting aspect. Electronic handover systems have the potential to improve interprofessional communication and thus healthcare for employees with CMD. With the help of an electronic handover system, information could be shared in a straightforward, quick, and reliable manner. This study provides insight into the desired content and functional requirements by psychotherapists, OPs and CIM members. Several additional steps are necessary for a further theoretical and practical development of an electronic handover system. For this purpose, other relevant stakeholders such as patients, data protection officers and software developers need to be involved. Furthermore, it needs to be examined to what extent the requirements could be implemented in already existing electronic systems (e.g. EHRs).

## Supplementary Information

**Additional file 1.** Topic guide.

**Additional file 2.** Completed checklist of the consolidated criteria for reporting qualitative research (COREQ).

## Data Availability

The datasets used and/or analysed in this study cannot be handed out due to legal data protection requirements.

## Abbreviations

CMD: Common mental disorders
OPs: Occupational physicians
CIM: Company integration management
EHR: Electronic health record
COREQ: Checklist of consolidated criteria for reporting qualitative research
Q: Quote
