# Supplementary material for: Requirements for an electronic handover system for interprofessional collaboration between psychotherapists and occupational health professionals – a qualitative study
Source: BMC Health Serv Res. 2022 Aug 25;22:1087. doi: 10.1186/s12913-022-08381-9 (PMC9403231; doi:10.1186/s12913-022-08381-9)
Supplement: Supplementary file 1 — Additional file 1. Topic guide. [file 12913_2022_8381_MOESM1_ESM.pdf]

Topic guide for focus groups

Aims:

- To create a catalogue of requirements for an electronic handover system to exchange patient-related information

Target group:

- Occupational Physicians
- Psychotherapists (ambulant, stationary or working in rehab hospitals)
- Members of a company integration management team (CIM team)

1) Introduction

a. Welcoming
b. Brief, mutual introduction of all participants (occupational group, functions)
c. Data protection/confidentiality, technical procedure

We collect all information pseudonymously. Therefore, we assigned a number to each of you. When we start recording, you can use these numbers to refer to each other. So please, do not use any names, and if it does happen, these parts will be redacted in the transcription. Confidentiality of your data is maintained at all times. This means that no conclusions concerning you or your employer can be drawn, neither in the final report nor in publications. Ms. Kohl will take additional notes during the session that will help us with the transcription and analysis later on.

Please feel free to simply start talking, but there is also a function in WebEx where you can virtually raise your hand. If you click on the three buttons in your bar, you should have the option “Raise Hand”. This will then appear in the list of participants, which you can unfold on the right side of the screen. I am going to pay attention to that during the discussion round and call on you. After that, you can lower your hand by clicking the same button. Can all of you see this function?

Besides, we simply want to hear about your experiences and opinions during this discussion. In the course of the conversation, I will ask you some open questions and I would like you to discuss anything relevant and important.

To avoid background noises, we would like you to switch your microphone off as long as you are not speaking.

d. Give a short overview of the study’s backgrounds and the aim of the focus groups

Before we start our discussion, I would like to briefly summarise our project for you. Ms. Kohl and I work in the FRIAA project. This is a larger collaborative project with other universities and institutions, in which we would like to test a concept of psychosomatic consultation hours in the workplace within a randomised controlled trial. We mainly want to focus on early detection and early intervention of mental problems among employees. Employees with mental problems and mental illnesses can be referred to the psychotherapeutic consultation hours by their occupational physicians, by supervisors or by themselves. Employees always get a diagnostic consultation there and up to ten further psychotherapeutic sessions if needed. The special feature is that work-related aspects are explicitly addressed in this consultation, as well. The intervention does not only contain early detection and early intervention but also has a module in which employees who were absent for a longer time due to mental illnesses can receive psychotherapeutic assistance in the process of company reintegration. In all modules, it is important for us to also include occupational physicians and the CIM team, or rather to reinforce communication between occupational physicians, the CIM team and psychotherapists. This should be accomplished with the help of an electronic handover system, thus an electronic patient file.

Do you have any more questions?

e. Start recording

We would then like to start recording and ask you to refer to yourselves and each other with the different numbers.

2) *Introductory question on occupational-medical courses AND patient-related collaboration between occupational physicians, therapists with medical or psychological training from the ambulant or stational field (acute or rehabilitative):*
   Have you contacted other occupational groups in the care and treatment of employees with mental illnesses in the past?
   a. How did you experience this collaboration?
   b. Which beneficial and impeding aspects were significant during collaboration? (In case of no collaboration: Which aspects kept you from collaborating?)
3) Introductory question concerning an electronic handover system for the exchange of patient-related data: Try to think of situations in which you contacted people from other occupational groups in the care and treatment of patients with mental illnesses. Which information did you exchange, or did you want to exchange? [If the previous discussion showed that there has not been any exchange between the individual stakeholders yet: Would you appreciate an exchange with the other occupational groups in the future and if so, what would you like to exchange?]
4) Key questions concerning an electronic handover system
   a. Please imagine that there is an electronic handover system to exchange patient-related data that could be used to inform psychotherapists, occupational physicians, other physicians involved and the CIM team during the treatment and care of employees with mental illnesses. Medical confidentiality would be maintained at all times, and the patients themselves could decide who is allowed to get access to which information. What requirements would you have for such an electronic handover system in the treatment and care of employees with mental illnesses?
   b. What kind of content should be shared via an electronic handover system with whom and how detailed should the content be? [If necessary, ask for contents that have already been mentioned in the literature: information on workplace and workplace conditions, possibilities of change at work (for example shift change), diagnostic findings, utilised measuring methods, including results, discharge papers]
      i. What kind of information do you need from other occupational groups, and how detailed should the information be?
      ii. What kind of information would you like to share with other occupational groups? How detailed would the information be?
      iii. What kind of information would you like to receive from the patient's employer?
      iv. What kind of information would you like to share with the employer?
      v. Do you think that there are other stakeholders that should be included in such an electronic handover system?
   c. In which situations, or when in the course of therapy, would you use such an electronic handover system and for what?
   d. What other functions should a handover system have? [If necessary, ask for further clues like functions to upload documents and medical findings, free text functions, chat functions, time requirement]
   e. In your opinion, what should the system have in terms of operability?
   f. Which factors would keep you from using an electronic handover system?
5) Final question on electronic handovers: In your opinion, is there anything else that should be considered when developing a handover system?
