# Supplementary material for: Requirements for an electronic handover system for interprofessional collaboration between psychotherapists and occupational health professionals – a qualitative study
Source: BMC Health Serv Res. 2022 Aug 25;22:1087. doi: 10.1186/s12913-022-08381-9 (PMC9403231; doi:10.1186/s12913-022-08381-9)
Supplement: Supplementary file 2 — Additional file 2. Completed checklist of the consolidated criteria for reporting qualitative research (COREQ). [file 12913_2022_8381_MOESM2_ESM.pdf]

Completed checklist of the "Consolidated criteria for reporting qualitative studies (COREQ): 32-item checklist"[1]

No	Item	Guide question/description
**Domain 1: Research team and reflexivity**		
Personal characteristics		
1.	Interviewer/facilitator	Jeannette Weber and Fiona Kohl
2.	Credentials	(FK) Fiona Kohl (MPH), (JW) Dr. PH Jeannette Weber, (PA) Prof. Dr. Peter Angerer, Lisa Guthardt (LG)
3.	Occupation	FK: research associate, JW: research associate, LG: research associate, PA: university professor
4.	Gender	FK, JW and LG female; PA male
5.	Experience and training	FK: educational background in physiotherapy and public health, practical experience in occupational health research, occupational health promotion, diabetes research and physiotherapy; JW: educational background in biomedical science and public health, practical experience in occupational health research and qualitative research; LG: educational background in English translation, PA: educational background in human medical studies, experience as a clinical doctor and in occupational health research
Relationship with participants		
6.	Relationship established	FK and JW knew some participants on a personal or professional basis.
7.	Participant knowledge of the Interviewer	Participants knew that JW and FK were working as research associates at the Institute for Occupational, Social and Environmental Medicine of the Heinrich-Heine-University Düsseldorf, Germany. Since these participants were treated in the same way as the others and had no prior knowledge of the subject of the study, no bias in the study results can be assumed in this regard.
8.	Interviewer characteristics	No other characteristics were reported
**Domain 2: study design**		
Theoretical framework		
9.	Methodological orientation and Theory	Methods of qualitative content analysis by Mayring. Deductive categories: (1) content-related requirements and (2) functional requirements. Content-related requirements were further deductively categorised in (i) information for occupational physicians, (ii) information for psychotherapists, and (iii) information for CIM members. These overarching categories were subdivided into subcategories by inductive coding.
Participant selection		
10.	Sampling	Purposive sampling by using predefined criteria. Efforts were made to reach participants with experience from different areas of relevant professional fields as well as from different regions of Germany.
11.	Method of approach	Via personal contact and education course for occupational medicine
12.	Sample size	25 participants
13.	Non-participation	Study was approached to 81 people, 32 agreed to attend and 25 participated in focus groups.
Setting		
14.	Setting of data collection	Online video conference via Cisco WebEx
15.	Presence of non-participants	FK or Susan Gritzka (SG) took field notes
16.	Description of sample	Psychotherapists: median age = 54 (range: 33-67 years), female = 44%<br>OPs: median age = 57 (range: 36-68 years), female = 82%<br>CIM members: median age = 49 (range: 42-61 years), female = 100%
Data collection		
17.	Interview guide	Provided as supplemental material. The subject guide was translated into English by LG.
18.	Repeat interview	None
19.	Audio/visual recording	Audio recording
20.	Field notes	Yes. Field notes were used in the analysis to consider internet connection failures and to match content to transcripts.
21.	Duration	App. 90 minutes
22.	Data saturation	Yes. Data saturation in statements from CIM members could not be achieved. Since most of the company physicians were also CIM members, it can nevertheless be assumed that data saturation was also achieved here.
23.	Transcripts returned	No due logistic constraints
**Domain 3: analysis and findings**		
24.	Number of data coders	Two
25.	Description of the coding tree	Provided in figure 1 of the study
26.	Derivation of themes	Deductive categories: (1) content-related requirements and (2) functional requirements. Content-related requirements were further deductively categorised in (i) information for occupational physicians, (ii) information for psychotherapists, and (iii) information for CIM members. These overarching categories were subdivided into subcategories by inductive coding.
27.	Software	MAXQDA 2018
28.	Participant checking	No
Reporting		
29.	Quotations presented	Yes. For content requirements in table 2 and structural requirements in table 3
30.	Data and findings consistent	Yes

31.	Clarity of major themes	Yes
32.	Clarity of minor themes	Yes

1. Tong, A., P. Sainsbury, and J. Craig, *Consolidated Criteria for Reporting Qualitative Research (COREQ): A 32-Item Checklist for Interviews and Focus Groups.* International journal for quality in health care : journal of the International Society for Quality in Health Care / ISQua, 2008. **19**: p. 349-57.
